# Mechanistic Elucidation of Activation/Deactivation Signal Transduction within Neurotensin Receptor 1 Triggered by ‘Driver Chemical Groups’ of Modulators: A Comparative Molecular Dynamics Simulation

**DOI:** 10.3390/pharmaceutics15072000

**Published:** 2023-07-21

**Authors:** Xun Lu, Xinchao Shi, Jigang Fan, Mingyu Li, Yuxiang Zhang, Shaoyong Lu, Guanghuan Xu, Ziqiang Chen

**Affiliations:** 1 Medicinal Chemistry and Bioinformatics Center, Shanghai Jiao Tong University School of Medicine, Shanghai 200025, Chinalushaoyong@sjtu.edu.cn (S.L.); 2 Department of VIP Clinic, Changhai Hospital, Affiliated to Navy Medical University, Shanghai 200433, China; 3 Department of Orthopedics, Changhai Hospital, Affiliated to Naval Medical University, Shanghai 200433, China

**Keywords:** neurotensin receptor 1, molecular dynamic simulation, signal transduction, selective interaction

## Abstract

Small-molecule modulators of neurotensin receptor 1 (NTSR1), a class A G-protein-coupled receptor (GPCR), has emerged as promising therapeutic agent for psychiatric disorders and cancer. Interestingly, a chemical group substitution in NTSR1 modulators can launch different types of downstream regulation, highlighting the significance of deciphering the internal fine-tuning mechanism. Here, we conducted a synergistic application of a Gaussian accelerated molecular dynamics simulation, a conventional molecular dynamics simulation, and Markov state models (MSM) to investigate the underlying mechanism of ‘driver chemical groups’ of modulators triggering inverse signaling. The results indicated that the flexibility of the leucine moiety in NTSR1 agonists contributes to the inward displacement of TM7 through a loosely coupled allosteric pathway, while the rigidity of the adamantane moiety in NTSR1 antagonists leads to unfavorable downward transduction of agonistic signaling. Furthermore, we found that R322^6.54^, Y319^6.51^, F353^7.42^, R148^3.32^, S356^7.45^, and S357^7.46^ may play a key role in inducing the activation of NTSR1. Together, our findings not only highlight the ingenious signal transduction within class A GPCRs but also lay a foundation for the development of targeted drugs harboring different regulatory functions of NTSR1.

## 1. Introduction

G-protein-coupled receptors (GPCRs) are the most abundant type of receptors on eukaryotic cell membranes. Each of them typically contains a conversed architecture of seven transmembrane helices (TMs) that divide the receptor into the N-terminus, C-terminus, three extracellular loops (ECLs), and three intracellular loops (ICLs) [1]. Once anchored by specific ligands, the receptor is activated through conformational rearrangements, followed by the recruitment of corresponding G proteins or β-arrestins to the intracellular binding site, triggering downstream signaling [2].

Neurotensin receptor 1 (NTSR1), a prototypical class A GPCR, plays a prominent role in the central nervous system and the periphery [3]. In the past 40 years, researchers have extensively developed small molecules and peptides to explore its biological functions [4,5,6,7,8,9]. These modulators, according to their biological effect, can be categorized into full agonists, partial agonists, and antagonists [10]. Further studies have shown that NTSR1 agonists play an anti-addictive role in the central nervous system, while antagonists inhibit the invasion and migration of peripheral cells, demonstrating the potential to treat a variety of peripheral tumors [3].

**Figure 1:**
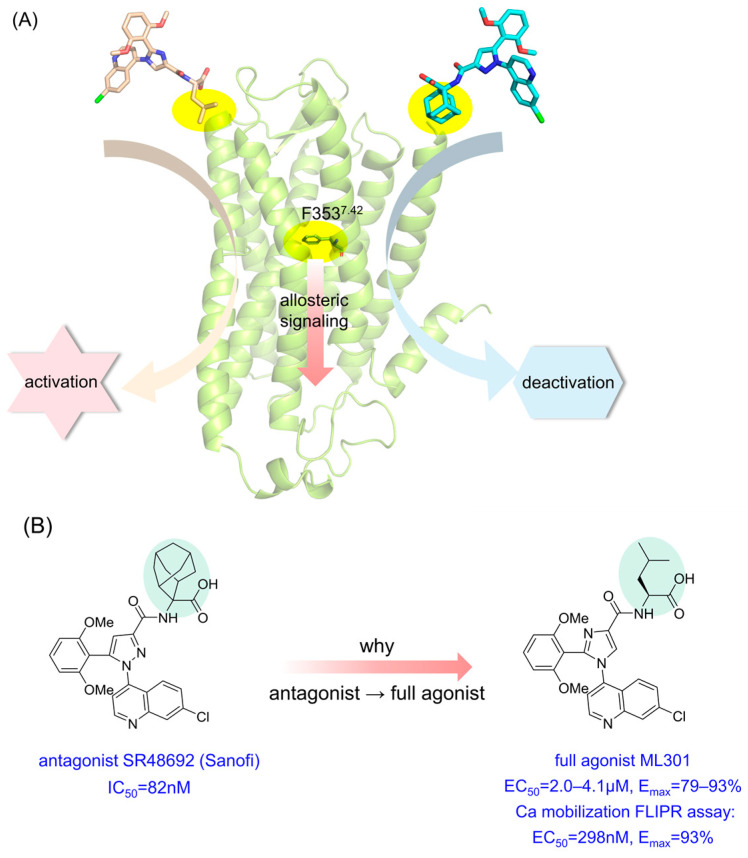
Graphic abstract of one ‘driver chemical group’ triggering inverse downstream signaling. (**A**) The abridged general view of NTSR1 antagonist SR48692 and full agonist ML301 inducing activation/deactivation signal transduction via the switch function of F353^7.42^. (**B**) The structure and biological activity of SR48692 and ML301.

Among modulators, a ubiquitous phenomenon is that merely the substitution of adamantane into the leucine moiety reverses antagonists into full agonists [6] (Figure 1). Mechanistic studies of ‘driver chemical group’ phenomena [11] like this will be conducive to fine-tuning signal regulation within class A GPCRs and accelerating the design of NTSR1 agonists or antagonists.

Recently, the complexes of NTSR1 and its ligands have been determined by either crystal or cryo-electron microscopy (cryo–EM) methodologies [12,13,14,15,16], laying solid underpinning for this research. However, these snapshots are intrinsically static and thus not sufficient to represent the dynamics of conformational ensembles to explain differences in protein internal signaling. Molecular dynamics (MD) simulations initiated from static crystallographic structures simulate dynamical information on the atomic level and conformational transitions, serving as a crucial complementation for crystallography [17,18]. Their biophysical application ranges from protein conformational studies [19,20] and allosteric mechanisms [21,22] to drug discovery [23,24,25,26].

In view of the conformational transition of GPCRs occurring over a large time scale, one of the enhanced sampling methodologies, Gaussian accelerated molecular dynamics (GaMD) simulation, has been employed. GaMD adds a harmonic boost potential, which follows a near-Gaussian distribution, to smoothen the potential energy surface of the system, accelerating the transition between low-energy states [27]. With the advantage of not needing to set predefined reaction coordinates or reduce the energetic noise, GaMD has witnessed extensive applications in GPCR conformational exploration [28,29].

Here, we selected the representative NTSR1 agonist ML301 and the antagonist SR48692 to decipher the mechanism of one-group difference triggering inverse NTSR1 signaling. GaMD (a total of 12 μs) was performed as a pioneer to broaden the conformational landscape, while the synergistic application of conventional MD (cMD) simulation (a total of 10.5 μs) and Markov state models were conducted to characterize the detailed conformational dynamics of NTSR1 in different states. The results indicated that the flexibility distinction of the leucine/adamantane moiety contributes to different signal transduction via a loosely coupled allosteric network. Furthermore, we found that R322^6.54^, Y319^6.51^, F353^7.42^, S356^7.45^, and S357^7.46^ might play a constructive role in inducing the activation of the NTSR1 receptor. Collectively, this research provides dynamic insights into the elaborate signaling pathway within NTSR1 and lays a promising foundation for the refinement of modulators harboring different regulatory functions of the NTSR1 receptor.

## 2. Materials and Methods

### 2.1. Construction of Stimulated Systems

Four model systems were built for MD simulations: an inactive NTSR1 + ML301 system, an inactive NTSR1 + SR48692 system, an active NTSR1 + ML301 system, and an active NTSR1 + SR48692 system. The inactive NTSR1 structure was obtained by homology modeling based on the corresponding murine structure (PDB ID: 6ZIN) [15]. SWISS-MODEL (https://swissmodel.expasy.org/ (accessed on 21 May 2018)) [30] and Pymol were utilized to remodel the truncated loops, carry out homology modeling, and remove non-NTSR1 co-crystallized molecules. The modeled structure was optimized using 100 ns cMD simulation and then validated by the Ramachandran Plot [31,32], ERRAT [33], VERIFY-3D [34,35], ProSA [36], and ProQ [37] programs (Figure S1). The obtained inactive state structure was then used as a receptor for the molecular docking of ML301 and SR48692 utilizing Autodock Vina. The Kollhman charges on the protein were calculated to be 23.5, and the Gasteiger charges on the ligands were −1.0002. The grid box size was set to 20 Å in three dimensions, while the x, y, and z grid box coordinates were set to (−0.35, −1.19, −18.42). The significant interaction between the carboxyl moiety of the ligand and R322^6.54^ was taken into account. Ten conformations of the ligand were written out as the output. The output ligand poses were carefully aligned since adamantane versus leucine is the only major difference between the two compounds. Using the canonical state NTSR1 (PDB ID: 6OS9) [13] as the receptor, the active NTSR1 + ML301 complex and the active NTSR1 + SR48692 complex were constructed in a similar way. The Kollhman charges on the protein and the Gasteiger charges on the ligands were 23.5 and −1.0009, respectively. The grid box size was set to 20 Å in three dimensions, while the x, y, and z grid box coordinates were set to 112.06, 136.95, and 139.85. The interaction between the carboxyl moiety of the ligand and R322^6.54^ were considered; 10 conformations of the ligand were written out as the output and then carefully chosen. Then, the obtained complexes were oriented in the Orientations of Proteins in Membrane (OPM) server (opm.phar.umich.edu/ (accessed on 2 September 2011)) [38] and inserted into the POPC membrane (the number of lipid molecules in the upper leaflet was 64, while the number of lipid molecules in the lower leaflet was 68) in the CHARMM-GUI server [39]. Next, the systems were embedded in TIP3P water molecules with a length of 10 Å. A counterion concentration of 0.15 mol/L KCl was used to balance the system charge [40]. Finally, we used the Amber tLeap program to generate the coordinate and topology files for the simulation with a lipid 14 force field for the POPC membrane [41], an ff14SB force field for proteins [42], a GAFF force field for ligands [43], and a TIP3P model for water molecules [44].

### 2.2. Gaussian Accelerated Molecular Dynamics (GaMD) Simulations

The systems were first minimized with a restraint of 500 kcal mol^−1^Å^−2^ on the NTSR1 receptors and ligands, while water and counterions were minimized in the 30,000 deepest descent cycles followed by 20,000 conjugate gradient cycles. Second, all atoms were subjected to 4000 cycles of the steepest descent and 20,000 cycles of conjugate gradient minimization without any restraints. Next, each system was gradually thermalized from 0 K to 310 K in 700 ps under isothermal–isovolumetric (NVT) conditions and finally equilibrated for 3.5 ns in an isothermal–isobaric (NPT) ensemble.

In the GaMD simulation method, when the system potential V(r) is lower than the reference energy E at position r, the updated V*(r) was calculated using Equations (1) and (2):

(1) $$V^{*}(\overset{⃑}{r})=V(\overset{⃑}{r})+∆V(\overset{⃑}{r})$$

(2) $$∆V(\overset{⃑}{r})=\left\{\begin{array}{c} {\frac{1}{2}k\left(E-V\left(\overset{⃑}{r}\right)\right)}^{2},\;V\left(\overset{⃑}{r}\right)<E\\ 0,\;V\left(\overset{⃑}{r}\right)\geq E \end{array}\right.$$

where the two parameters E and k (the harmonic force constant) were automatically adjusted using Equations (3) and (4):

(3) $$V_{max}\leq E\leq V_{min}+\frac{1}{k}$$

(4) $$k=k_{0}\frac{1}{V_{max}-V_{min}}$$

If E is set to the lower bound as $V_{max}$, then $k_{0}$ can be calculated by Equation (5), while if E is set to the upper bound $E=V_{min}+\frac{1}{k}$, then $k_{0}$ can be calculated by Equation (6):

(5) $$k_{0}=min(1.0,\;\frac{\sigma_{0}}{\sigma_{v}}\times \frac{V_{max}-V_{min}}{V_{max}-V_{avg}})$$

(6) $$k_{0}=(1.0-\frac{\sigma_{0}}{\sigma_{v}})\times \frac{V_{max}-V_{min}}{V_{avg}-V_{min}}$$

where $V_{max}$, $V_{min}$, and $V_{avg}$ denote the maximum, minimum, and averaged potential energy of the simulated systems, respectively, and $\sigma_{v}$ and $\sigma_{0}$ refer to the standard deviation of the potential energy and the user-specified upper limit for proper reweighting, respectively.

To conduct GaMD product simulation, a conventional MD simulation of 100 ns was first performed to obtain $V_{max}$, $V_{min}$, $V_{avg}$, and $\sigma_{v}$ and the greatest $\sigma_{0}$ and $k_{0}$. Next, 60 ns of GaMD equilibration were conducted to obtain the boost potential [27]. Lastly, four systems underwent 3 rounds of 1 μs dual-boost GaMD simulations with random velocities and an integration step of 2.0 fs. During the simulations, the particle mesh Ewald (PME) method was employed to evaluate the long-range electrostatic interactions, while a cutoff of 10 Å was used for short-range electrostatic and van der Waals interactions [45]. Covalent bonds involving hydrogen were restricted using the SHAKE algorithm [46]. The temperature of the systems was kept at 310 K using Langevin dynamics with the coupling time constant of 1.0 ps. The coordinates of the snapshots were collected every 200 ps.

### 2.3. Conventional Molecular Dynamics (cMD) Simulations

To reasonably select replicas in the GaMD simulations, the K-means clustering algorithm was performed, and finally, two replicas of the active NTSR1 + ML301 system and five replicas of the active NTSR1 + SR48692 system were selected. The restart files were extracted according to the representative frames for the input of the cMD simulation. Then, seven systems underwent 3 rounds of 500 ns cMD simulations with random velocities and an integration step of 2.0 fs. The simulation settings and methods were consistent with the GaMD simulations, except the boost potential was removed.

### 2.4. Dynamic Cross-Correlation Matrix (DCCM) Analysis

A Dynamic Cross-Correlation Matrix (DCCM) analysis was performed using the CPPTRAJ module [47] of AMBER18 on representative trajectories to investigate the coupled motions between atoms. According to the normalized cross-correlation matrix C, the ‘Pearson-like’ cross-correlation coefficient () was calculated using Equation (7):

(7) $$C_{ij}=\frac{\langle {(r}_{i}-\langle r_{i}\rangle \rangle \cdot \langle {(r}_{j}-\langle r_{j}\rangle \rangle }{\sqrt{{\langle {(r}_{i}-\langle r_{i}\rangle \rangle }^{2}\cdot {\langle {(r}_{j}-\langle r_{j}\rangle \rangle }^{2}}}$$

where $r_{i}$ and $r_{j}$ indicate the position vectors of the ith and jth atoms.

### 2.5. Principal Component Analysis (PCA) and Free Energy Landscape (FEL)

To capture the dominant motions during simulation, an effective statistical method, PCA, was introduced by constructing a covariance matrix, diagonalizing the matrix to generate eigenvectors, and computing the eigenvalues on the basis of the mean square fluctuation of trajectories projected along the eigenvectors. The eigenvectors, interpreted as principal components, were ranked by eigenvalues, with the top-ranked eigenvectors (such as PC1 and PC2) representing the most influential dynamics of the system [48]:

(8) $$G_{i}=-k_{B}Tln(\frac{N_{i}}{N_{m}})$$

where $k_{B}$, T, $N_{i}$, and $N_{m}$ represent Boltzmann constant, the simulation temperature, the population of the ith bin, and the population of most populated bins, respectively. To enhance the diversity of the indicator selection to ensure a comprehensive conformational depiction, we selected indicators, including the distance, angle, and RMSD values.

### 2.6. Community Network Analysis (CNA)

Benefiting from the correlation coefficient matrix $C_{ij}$ and the NetworkView plugin in VMD, we computed the community organization distinction between the active NTSR1 + ML301 system and the active NTSR1 + SR48692 system. In this analysis, each $C_{\alpha }$ atom was recognized as a node. On the basis of Equation (7), the edge connections between nodes were further calculated using Equation (9):

(9) $$d_{i,j}=-log(|C_{i,j}|)$$

where $C_{ij}$ was computed using Equation (7); i and j represent two nodes here. Two nodes are considered connected with a cutoff distance of 4.5 Å for at least 75% of the simulation time. Next, connected substructures, namely ‘communities’, were generated utilizing the Girvan−Newman algorithm with a cutoff residue number of 3. The edge betweenness, defined as the number of optimal paths traveled across a certain edge, was then calculated and set to be proportional to the width of the bonds bridging communities [49,50].

### 2.7. Markov State Models (MSM) Construction and Validation

Harnessing activation/deactivation parameters as the input, Markov state models (MSMs) were constructed following the standard PyEMMA protocol (http://www.emma-project.org/latest/ (accessed on 14 October 2015)) [51]. First, both the active NTSR1 + ML301 system and the active NTSR1 + SR48692 system were validated as Markovian through implied timescale (ITS) verification obeying Equation (10):

(10) $$t_{i}=\frac{-\tau }{ln|\lambda_{i}(\tau )|}$$

where τ represents the lag time and $\lambda_{i}$ denotes the eigenvalues of the Markov transition matrix.

Then, the free-energy landscape was decomposed into 120 microstates using the K-means clustering algorithm, and MSMs were established with an ITS-unaffected lag time of 5 ns. Thereupon, the Perron Cluster Analysis (PCCA+) algorithm was assigned to converge the microstates into four metastates, and the Chapman–Kolmogorov test was subsequently conducted to validate them as Markovian. Next, the transition path theory (TPT) was applied to calculate the mean first passage time (MFPT) for the activation/deactivation process on the basis of the transition probability matrix of the MSMs. Finally, utilizing the MDTraj package, we extracted the structures near the microstate cluster centers of the corresponding metastates into new trajectories and selected the representative conformation of each metastate according to the similarity score $S_{ij}$ given by Equation (11):

(11) $$S_{ij}=e^{-d_{ij}/d_{scale}}$$

where $d_{ij}$ is the RMSD between conformations *i* and *j*, and $d_{scale}$ is the standard deviation of *d*.

## 3. Results

### 3.1. Antagonist SR48692 and Agonist ML301 Binding Induce Respective Inactive and Active Conformations of NTSR1

To comprehensively explore the conformational landscape, each of the three 1 μs rounds of GaMD simulation was performed on four systems: the inactive NTSR1 + SR48692 system, the inactive NTSR1 + ML301 system, the active NTSR1 + SR48692 system, and the active NTSR1 + ML301 system. We defined two parameters (collective variables and CVs) to project the simulated trajectories onto a two-dimensional (2D) space to depict the global conformational transition during NTSR1 activation/deactivation. Since the most quintessential feature shared by class A GPCR activation is the outward movement of TM5 and TM6 and the inward displacement of TM7 on the intracellular side [19,20,32], the first CV was the distance between the center of mass of S253^5.55^ (superscripts indicate the Ballesteros–Weinstein numbering for GPCR residues) in TM5 and S356^7.45^–S357^7.46^ in TM7 (TM5–TM7 distance). The decrease in the TM5–TM7 distance represented the inward movement of TM7. The distance between the center of mass of Y103^2.41^ in TM2 and V302^6.34^ in TM6 (TM2–TM6 distance) was defined as the second CV. The increase in the TM2–TM6 distance represented the outward displacement of TM6.

**Figure 2:**
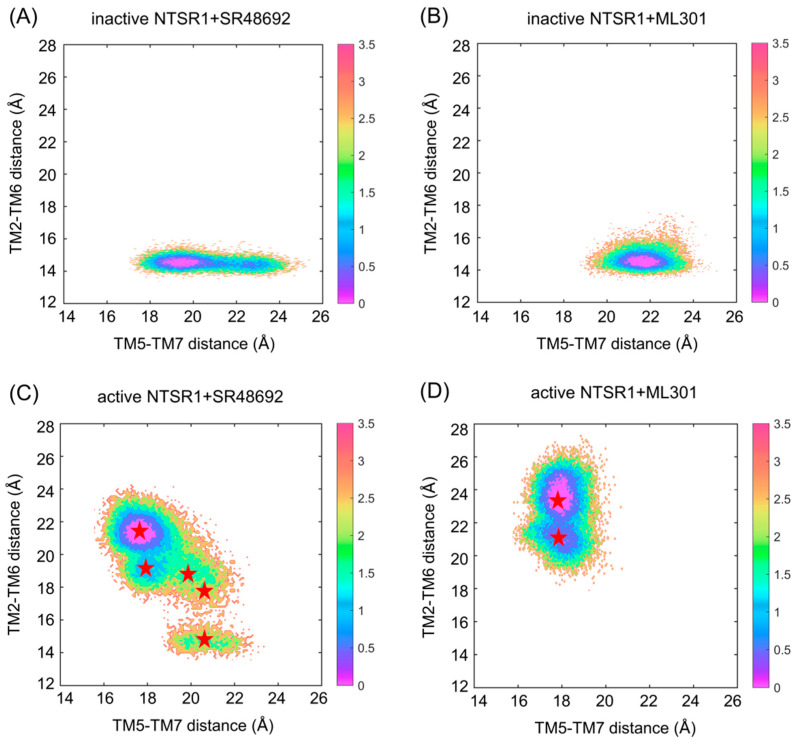
The free energy landscapes of the inactive NTSR1 + SR48692 system (**A**), the inactive NTSR1 + ML301 system (**B**), the active NTSR1 + SR48692 system (**C**), and the active NTSR1 + ML301 system (**D**) are shown by simulation trajectory projection. Collective variable 1 (CV1): TM5–TM7 distance; CV2: TM2–TM6 distance. The color scales on the right were evaluated through density. The red stars denote the positions of representative structures extracted using K-means clustering.

Antagonist SR48692 and agonist ML301 binding induced a distinct conformational ensemble in NTSR1 (Figure 2). Since the initial inactive structure was characterized by a TM5–TM7 distance of 22.45 Å and a TM2–TM6 distance of 14.42 Å, the density basins with a TM5–TM7 distance of ~20–23 Å and a TM2–TM6 distance of ~14–15 Å represented the inactive state (Figure 2A,B). Similarly, with the initial active structure featuring a TM5–TM7 distance of 16.86 Å and a TM2–TM6 distance of 24.35 Å, the active conformation could be characterized by the density basin with a TM5–TM7 distance of ~16.5–19 Å and a TM2–TM6 distance of ~20.5–25 Å (Figure 2C,D). These observations suggest that the active conformations were inaccessible when initiated from the inactive state of NTSR1 due to the high energy barrier, even with agonist ML301 binding (Figure 2A,B), while the inactive conformations were captured when initiated from the active state of NTSR1 in the presence of antagonist SR48692 (Figure 2C,D). Once bound to SR48692, the receptor transited from the active state to the inactive state through several intermediate conformations (Figure 2C). By contrast, full agonist ML301 binding stabilized the receptor in the active state (Figure 2D). The 2D landscape projected by the other parameters (CV1: TM3–TM6 distance, evaluated by the distance between the center of mass of R166^3.50^ in TM3 and V302^6.34^ in TM6; CV2: NPxxY RMSD, evaluated by root mean square deviation of the non-symmetric side-chain atoms of residues N360^7.49^ to Y364^7.53^) demonstrated similar results (Figure S2).

**Figure 3:**
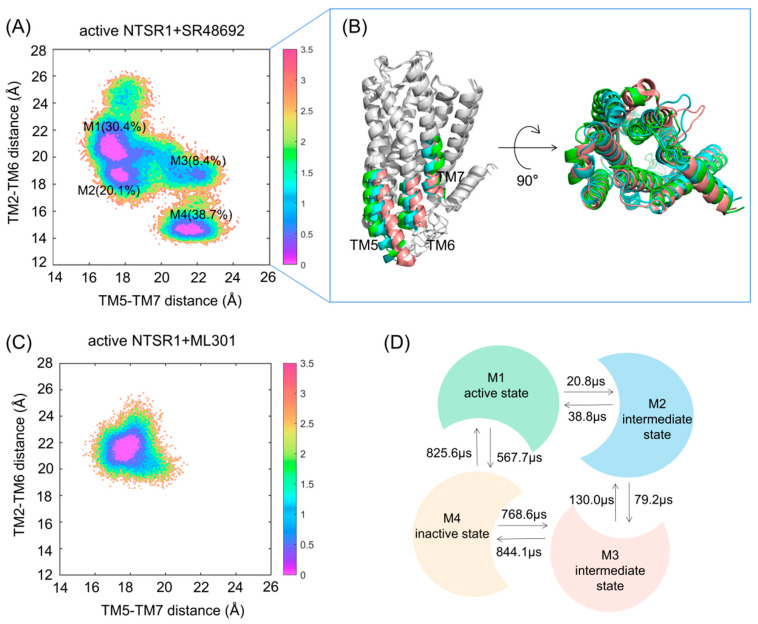
The free energy landscapes of the active NTSR1 + SR48692 system (**A**) and the active NTSR1 + ML301 system (**C**) in cMD simulation. The units of free energy values are kcal/mol. The color scale on the right was evaluated through free energy. (**B**) The representative conformations of the active NTSR1 + SR48692 system in cMD simulation. Active state, tangerine; intermediate state, cyan; inactive state, green. (**D**) The transition timescale among representative conformations of the active NTSR1 + SR48692 system, represented by the mean first passage time.

We used K-means clustering to extract five representative conformations from the GaMD trajectories of the active NTSR1 + SR48692 system (Figure 2C) and two representative conformations from the GaMD trajectories of the active NTSR1 + ML301 system (Figure 2D), and we then performed an additional three 500 ns rounds of cMD simulations on the seven systems with each extracted conformation as the initial structure. The RMSD values of the ligands in all simulations were first proven to reach convergence in both systems (Figure S3A) in order to verify the rationality of our docking and simulation. Then, the free energy landscape was depicted using identical CVs. Antagonist SR48692 binding induces the gradual deactivation of NTSR1 through a transition pathway of M1 (the active state, 30.4%)→M2 (the intermediate state, 20.1%)→M3 (the intermediate state, 8.4%)→M4 (the inactive state, 38.7%) (Figure 3A). Significantly, the representative conformation extracted from each energy basin featured typical characteristics of the active state, the intermediate state, and the inactive state, as revealed by the conformational arrangements of TM5, TM6, and TM7 (Figure 3B). A porcupine plot was constructed to graphically visualize the dominant movements of different regions (Figure S3B). The outward shifts of TM5 and TM6 and the inward translocation of TM7 presented the dominant conformational dynamics despite the highly flexible loops. By contrast, agonist ML301 binding stabilized NTSR1 in the active state (Figure 3C). The 2D landscape projected by the TM3–TM6 distance (CV1) and NPxxY RMSD (CV2) illustrated similar results (Figure S3C,D).

We further applied Markov state models to unveil the transition detail of the active NTSR1 + SR48692 system (Figure 3D). The results indicated that the M1→M2 (20.8 μs) and M2→M3 (79.2 μs) transition times were shorter than the corresponding reverse processes (38.8 and 130.0 μs, respectively), which confirmed that the inactive state was more accessible than the active state with the binding of SR48692 to the active NTSR1. Notably, owing to the lowest population of the M3 state, a long timescale was required for the complete deactivation of the receptor, implying that NTSR1 deactivation is a slow process.

### 3.2. Agonist ML301 Binding Contributes to Enhanced Overall Conformational Dynamics and Distinct Domain Movement

**Figure 4:**
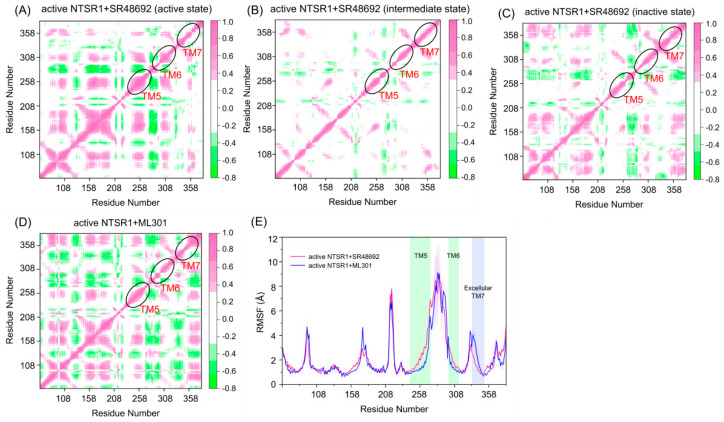
The dynamic cross−correlation matrix analysis of the active NTSR1 + SR48692 system (active state) (**A**), the active NTSR1 + SR48692 system (intermediate state) (**B**), the active NTSR1 + SR48692 system (inactive state) (**C**), and the active NTSR1 + ML301 system (**D**). Color scales are shown on the right. The interactions whose absolute correlation coefficients are less than 0.3 are colored white for clarity. (**E**) The RMSF analyses of the active NTSR1 + SR48692 system (pink curve) and the active NTSR1 + ML301 system (blue curve).

To reveal the dynamic movement of protein domains within the receptor, we conducted a dynamic cross-correlation matrix analysis using trajectories of representative conformations. In the active NTSR1 + SR48692 system, fewer correlations were observed in the active (Figure 4A), intermediate (Figure 4B), and inactive (Figure 4C) states. By contrast, enhanced movements were observed in the active NTSR1 + ML301 system (Figure 4D), thus altering the internal protein structure for enhanced signal propagation, which may promote activation signal transduction.

The atomic root-mean-square fluctuations (RMSFs) of Cα atoms around their original positions were subsequently quantified for each residue to compare the mobility of different regions (Figure 4E). Functional regions with major fluctuation in the two systems included TM5, TM6, and extracellular TM7. Due to the deactivation of the receptor, TM5 and TM6 of the active NTSR1 + SR48692 system displayed remarkable inward movement and thus fluctuated more frequently than those of the active NTSR1 + ML301 system. Notably, in the active NTSR1 + ML301 system, the extracellular region of TM7 experienced more fluctuation than that of the active NTSR1 + SR48692 system. Because the extracellular TM7 approached the ligand binding site, the residues within this region might function as a trigger for discriminating the activation or deactivation signal.

### 3.3. The Flexibility of Leucine Moiety in Agonist ML301 Contributes to the Inward Displacement of TM7

**Figure 5:**
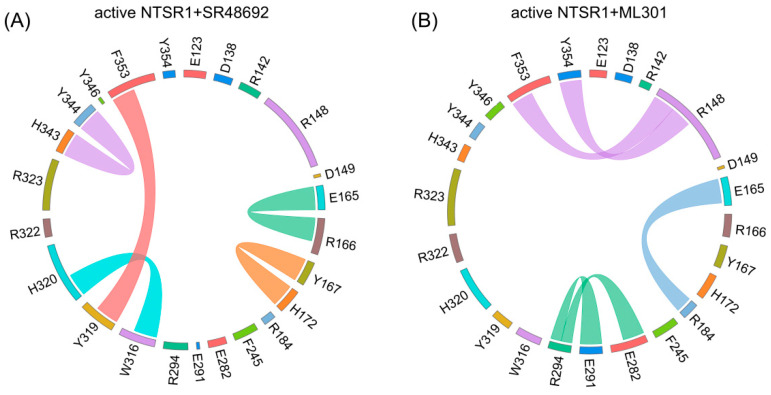
The specific interactions within the active NTSR1 + SR48692 system (**A**) and the active NTSR1 + ML301 system (**B**) revealed by proportional chord diagram. The chords connecting two residues denote specific interactions that occupy over 50% of the simulation time. In the two diagrams, the colors of chords correspond to specific interactions: green, salt bridge and sidechain-sidechain hydrogen bonds; orange, π-cation interaction and backbone–backbone hydrogen bonds; cyan, π-cation interaction, sidechain–sidechain hydrogen bonds and backbone–backbone hydrogen bonds; magenta, π-π stacking; purple, π-cation interaction; light blue, salt bridge.

The intramolecular interactions, including hydrogen bonds, salt bridges, polar interactions, and hydrophobic interactions, play critical roles in signal propagation within the protein [52]. To uncover distinct signal transduction, we analyzed different kinds of interactions in the active NTSR1 + SR48692 system and the active NTSR1 + ML301 system on the basis of the representative trajectories. Here, proportional chord diagrams were used to describe the specific interactions that occupied over 50% of the simulation time (Figure 5).

**Figure 6:**
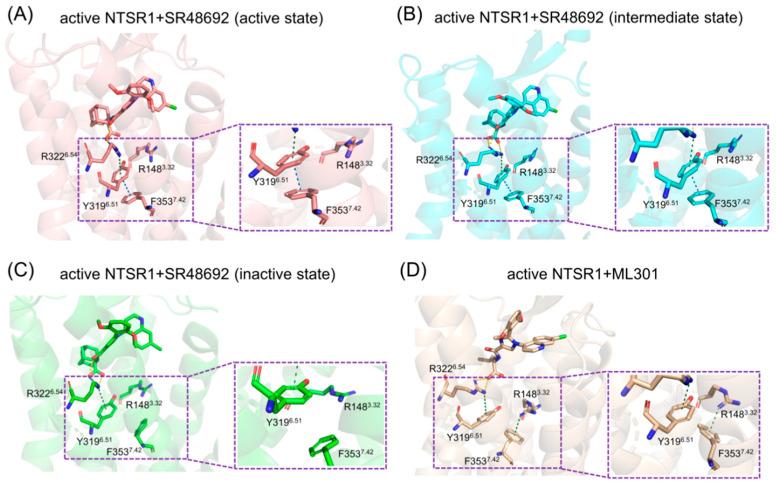
The interaction analysis of the active NTSR1 + SR48692 system (active state) (**A**), the active NTSR1 + SR48692 system (intermediate state) (**B**), the active NTSR1 + SR48692 system (inactive state) (**C**), and the active NTSR1 + ML301 system (**D**). Y319^6.51^-F353^7.42^-R148^3.32^ interactions are enlarged for clarity.

**Figure 7:**
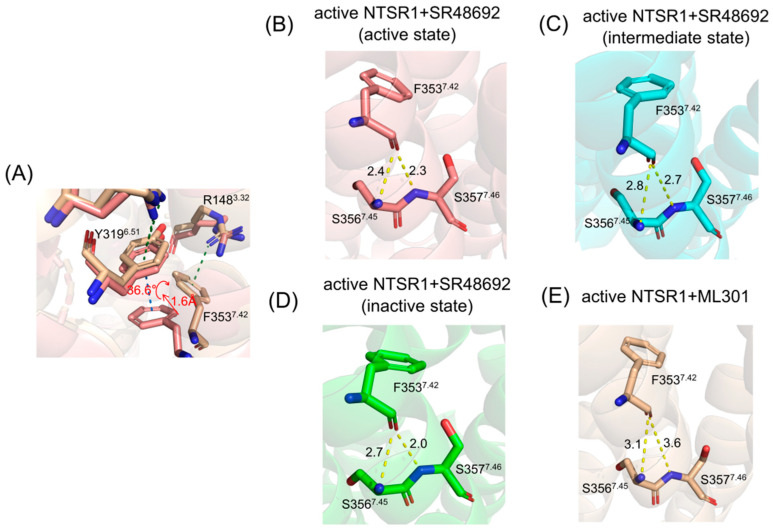
(**A**) The residue superimposed analysis of the active NTSR1 + SR48692 system (active state) (tangerine) and the active NTSR1 + ML301 system (wheat). The preference for hydrogen bonds among F353^7.42^-S356^7.45^/S357^7.46^ in the active NTSR1 + SR48692 system (active state) (**B**), the active NTSR1 + SR48692 system (intermediate state) (**C**), the active NTSR1 + SR48692 system (inactive state) (**D**), and the active NTSR1 + ML301 system (**E**).

Proximal to the ligand binding site, F353^7.42^ formed π-π stacking with Y319^6.51^ in the active NTSR1 + SR48692 system (Figure 5A). By contrast, it formed selective π-cation interactions with R148^3.32^ in the active NTSR1 + ML301 system (Figure 5B). During the simulations, we found that the adamantane moiety of antagonist SR48692 exhibited relative rigidity with less fluctuation (RMSF: 1.13 Å). The salt bridge between the ligand’s carboxyl group and R322^6.54^, the π-cation interaction between R322^6.54^ and Y319^6.51^, and the π-π stacking between Y319^6.51^ and F353^7.42^ were stable, and in which case the orientation of F353^7.42^ hindered its interaction with R148^3.32^ (Figure 6A–C). However, the leucine moiety of agonist ML301 experienced more fluctuation (RMSF: 1.22 Å) due to its flexibility, which could interact with Y319^6.51^ through the stable interaction of carboxyl group–R322^6.54^-Y319^6.51^. The vibration of Y319^6.51^ resulted in the breakage of its π-π stacking with F353^7.42^ (Figure 6D), thus driving F353^7.42^ to form a π-cation interaction with R148^3.32^ by forwarding 1.6 Å and rotating 36.6° (Figure 7A). Collectively, the flexibility of leucine moiety in agonist ML301 contributed to selective π-cation interaction between F353^7.42^ and R148^3.32^, which deciphers the first level of signal transduction.

In the second level of allosteric signaling, owing to the reorientation of F353^7.42^, the hydrogen bond preference for S356^7.45^/S357^7.46^ was reshaped. In the active NTSR1 + SR48692 system, the length of the hydrogen bond between F353^7.42^ and S357^7.46^ was shorter than that between F353^7.42^ and S356^7.45^, resulting in a preference for hydrogen bond formation between F353^7.42^ and S357^7.46^ (Figure 7B–D). By contrast, in the active NTSR1 + ML301 system, the right rotation of the aromatic ring caused the left rotation of the oxygen atom of F353^7.42^. As a result, the hydrogen bond preference experienced a transition from S357^7.46^ to S356^7.45^ (Figure 7E). Whereas S356^7.45^ and S357^7.46^ were located in the inward and outward regions of TM7, respectively, the transition of hydrogen bond preference ultimately resulted in rigidity release in the outward region of TM7 and thus urged its inward displacement. The displacement of TM7, a typical feature of class A GPCR activation, has a high potential to stabilize NTSR1 active conformation, which elucidates the agonistic activity of agonist ML301. Similarly, the hydrogen bond preference between F353^7.42^ and S357^7.46^ resulting from the adamantane moiety of antagonist SR48692 hinders the transduction of activation signals, so NTSR1 adheres to the intrinsic deactivation process of class A GPCRs [53].

### 3.4. Community Networks Indicate Preference for Activation Signal Transduction Originating from R148^3.32^

**Figure 8:**
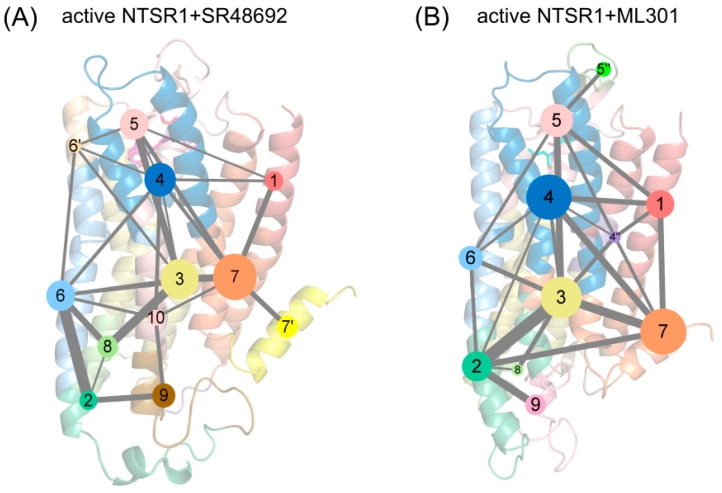
The community network analysis of the active NTSR1 + SR48692 system (**A**) and the active NTSR1 + ML301 system (**B**). Each sphere represents a corresponding community whose number of residue components is indicated by the sphere area. Each number denotes a community in the network, and number with a superscript refers to a sub-community. While the sticks connecting different spheres visualize the inter-community connections, and the thickness of these sticks is proportional to the value of edge connectivity.

The propagation of allosteric signals within NTSR1 was further explored using community network analysis to investigate the variational coupling among all communities. During the trajectory, residues within a cutoff distance of 4.5 Å for at least 75% of the simulation time were classified as part of the same communities, which were recognized as synergistic functional units within the overall protein. The visualized community network graphs provide clear depictions of the allosteric crosstalk paths and the corresponding intensities within NTSR1 in different systems (Figure 8).

Distinct alterations in the topological characteristics and the intercommunity communications within the NTSR1 allosteric network were observed with the binding of SR48692 and ML301. In the active NTSR1 + SR48692 system, the residues that engaged in the first level of signal transduction were involved in Community 4, which shared relatively weak and indirect interactions with Communities 2, 7, and 9 within the positions of intracellular TM5, TM6, and TM7 (Figure 8A). It is therefore hypothesized that such a weak correlation contributes to unfavorable downstream transduction of activation signals, in which case NTSR1 would exhibit a slow deactivation trend. However, in the active NTSR1 + ML301 system, R148^3.32^, which formed a selective π-cation interaction with F353^7.42^, belongs to Subcommunity 4″, which formed relatively strong interactions with Communities 2 and 7 (at the position of intracellular TM5, TM6, and TM7) mediated by Community 3 (Figure 8B). In view of this, it is implied that activation signals have a higher tendency to be transmitted to the intracellular TM5, TM6, and TM7.

Moreover, helix 8 of the active NTSR1 + SR48692 system belongs to Community 7′, independent of Community 7, so the gradual elimination of the membrane localization of helix 8, a canonical feature of class A GPCR deactivation, was more achievable (Figure 8A). Comparatively, in the active NTSR1 + ML301 system, helix 8 and TM7 jointly constitute Community 7, indicating the collaborative movement of the two domains and thus the stability of helix 8 membrane localization and the active conformation (Figure 8B).

## 4. Discussion

GPCRs are versatile cellular sensors for chemical stimuli, serving as promising targets for about 30% of approved drugs [54]. The prototypical class A GPCR NTSR1 exerts dual activity both in the central nervous system and the periphery, demonstrating brilliant therapeutic prospects. In the past several years, with the NTSR1 crystallographic complex with G-proteins and β-arrestins determined, the activation or deactivation regulation pathway within the receptor has been of intense interest. Furthermore, throughout decades of development of NTSR1 modulators, a ubiquitous phenomenon in the medicinal chemistry field has been that one single moiety difference in NTSR1 modulators can evoke distinct agonistic and antagonistic downstream signaling. For example, in full agonists SR12062 [8] and ML301 [10] and a partial agonist from the Research Triangle Institute (RTI) [6], substituting the leucine moiety for the adamantane moiety can transform the agonists into antagonists, like SR48692 [6] and SR-142948A [4]. Not fully interpreting the underlying mechanism of this phenomenon could inhibit drug discovery.

Some recent studies have made valuable efforts to explore the regulatory mechanism within NTSR1, but most of them have concentrated only on the activation/deactivation process induced by its endogenous peptide NTS or have employed machine learning to investigate the mechanism [55]. Considering the single engagement of NTS in mechanism exploration and the restrictions of machine learning in simulating and reproducing biological processes, the activation or deactivation regulation pathway within NTSR1 has not been explicitly decoded, hindering the understanding of the NTSR1 receptor and its downstream regulation.

Herein, to unravel the possible signal pathways within the receptor and further facilitate NTSR1 modulator discovery, we selected the NTSR1 full agonist ML301 and the antagonist SR48692 as representative modulators and performed GaMD simulation and cMD simulation, followed by Markov state models, DCCM, FEL, PCA, and CNA analysis. The results indicated that from a macroscopic perspective, the full agonist ML301 stimulated the NTSR1 internal structure to be more dynamic for activation signal propagation, with extracellular TM7 serving as the initiating region for discriminating the activation or deactivation signal. By stepwise dynamics exploration from a microscopic perspective, we uncovered that the flexibility of leucine indirectly accounted for selective π-cation interaction between F353^7.42^ and R148^3.32^, inducing the reorientation of F353^7.42^ and thus reshaping the hydrogen bond preference for S356^7.45^/S357^7.46^. It is implied that the hydrogen bond preference for S356^7.45^ subsequently results in the outward rigidity release and inward bending of TM7, thus contributing to the stability of the active conformation. Comparatively, the rigidity of adamantane moiety in the antagonist indirectly led to the hydrogen bond preference between F353^7.42^ and S357^7.46^, failing to block the intrinsic gradual deactivation process of class A GPCR. Such a loosely coupled allosteric network, comprising two main stages of signal transduction, links small perturbations at the extracellular ligand binding site to large conformational changes at the intracellular G-protein-binding site, explaining the cause of reverse biological effects induced by two modulators. Furthermore, R322^6.54^, Y319^6.51^, F353^7.42^, R148^3.32^, S356^7.45^, and S357^7.46^, in which F353^7.42^ functions as a junction switch, may play a constructive role in NTSR1 activation.

Delineating a mechanistic elucidation of inverse NTSR1 signaling, our comparative MD simulations provide guidance from both a chemical biology and a medicinal chemistry perspective. The key residues along the signaling pathway, if further validated using mutagenesis and BRET assay, can be deployed as a potential switch or controller for diverse regulatory processes and downstream effects. Furthermore, in drug design, compounds that adjust more deeply into the orthosteric pocket and interact with key residues like F353^7.42^ and R148^3.32^ may result in different NTSR1 activity by fine-tuning the orientation of residues and the corresponding interaction network. Hence, the directed design of NTSR1 agonists or antagonists may be achieved. Collectively, our investigation of the NTSR1 signaling pathway induced by ligands with one differing group enables a better understanding of the intrinsic regulation mechanism of NTSR1 and paves an avenue for NTSR1 modulator discovery. Very recently, a β-arrestin-biased allosteric modulator of NTSR1, namely SBI-553, and its corresponding co-crystallized structure [56] have been reported. In view of this breakthrough, more systematic investigations of the correlated pathway between orthosteric and allosteric sites will be appreciated in future research.

## Data Availability

Not applicable.

## Supplementary Materials

The following supporting information can be downloaded at: https://www.mdpi.com/article/10.3390/pharmaceutics15072000/s1, Figure S1: Validation of rationality of NTSR1 protein structure from homology modeling using Ramachandran Plot (A), ERRAT program (B), VERIFY3D program (C), ProSA program (D), and ProQ program (E); Figure S2: The analogical free energy landscape of the inactive NTSR1 + ML301 system (A), the inactive NTSR1 + SR48692 system (B), the active NTSR1 + SR48692 system (C), and the active NTSR1 + ML301 system (D) in GaMD simulation (CV1: TM3–TM6 distance, CV2: NPxxY RMSD); Figure S3: The free energy landscape of the active NTSR1 + SR48692 system (A) and the active NTSR1 + ML301 system (B) in cMD simulation (CV1: TM3–TM6 distance, CV2: NPxxY RMSD); (C) the RMSD value of ligands in all simulations in the active NTSR1 + SR48692 system and the active NTSR1 + ML301 system; (D) the principal pattern of motion of the active NTSR1 + SR48692 system; Figure S4: Implied timescale test for MSMs in the active NTSR1 + SR48692 system (A) and the active NTSR1 + ML301 system (C) at different lag times and Chapman–Kolmogorov test of metastable states for the active NTSR1 + SR48692 system (B) and the active NTSR1 + ML301 system (D). Table S1: Frequency of Y319^6.S51^–F353^7.42^ and F353^7.42^–R148^3.32^ interaction in the representative trajectories in the active NTSR1 + SR48692 system and the active NTSR1 + ML301 system.
