# Supplementary material for: Mechanistic Elucidation of Activation/Deactivation Signal Transduction within Neurotensin Receptor 1 Triggered by ‘Driver Chemical Groups’ of Modulators: A Comparative Molecular Dynamics Simulation
Source: Pharmaceutics. 2023 Jul 21;15(7):2000. doi: 10.3390/pharmaceutics15072000 (PMC10385606; doi:10.3390/pharmaceutics15072000)
Supplement: Supplementary file 1 [file pharmaceutics-15-02000-s001.zip › pharmaceutics-2489874-supplementary.pdf]

Supplemental Information

Mechanistic Elucidation of Activation or Deactivation Signal Transduction within Neurotensin Receptor 1 (NTSR1) Triggered by 'Driver Chemical Group': A Comparative Molecular Dynamics Simulation

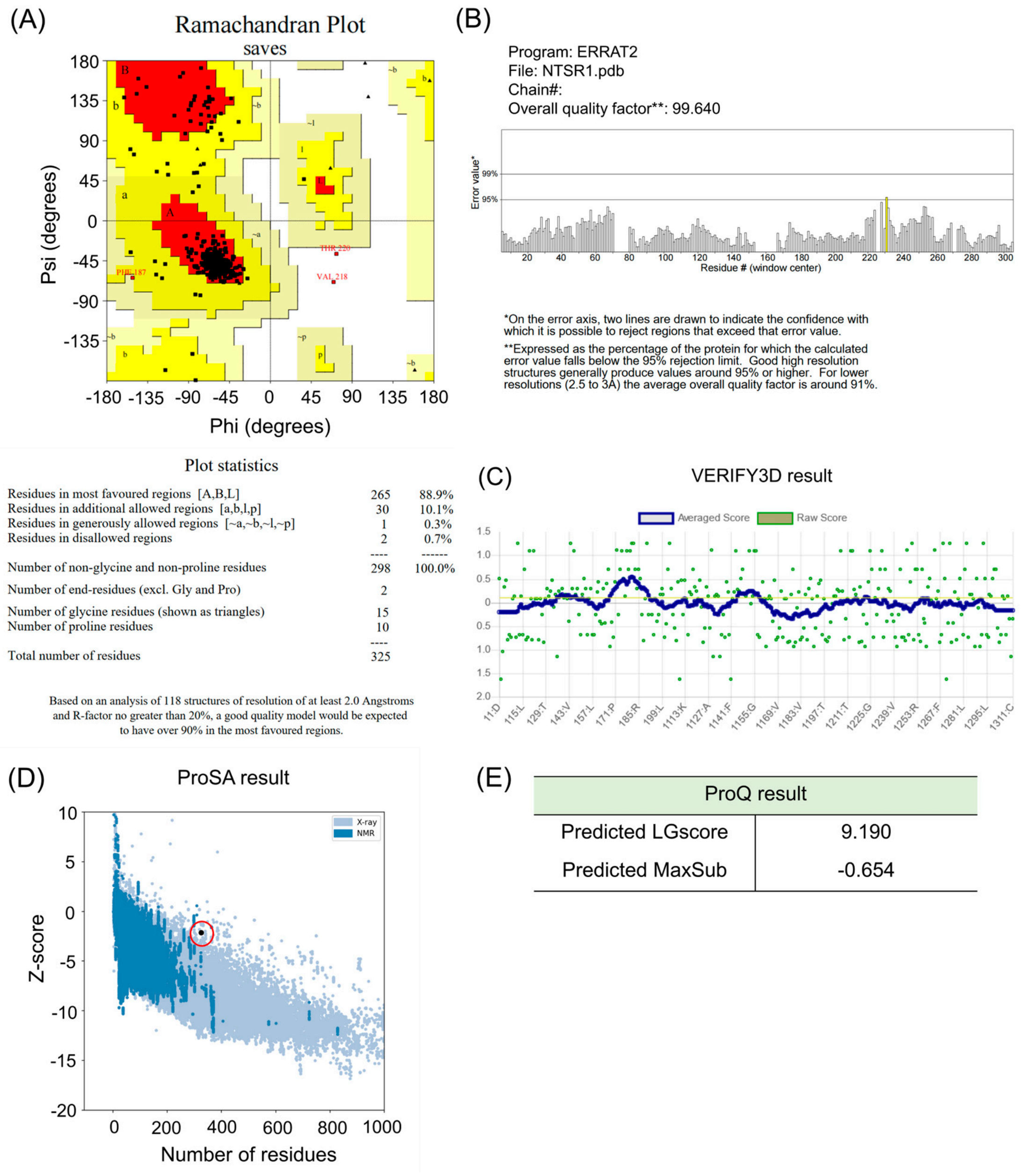

Figure S1. Validation of rationality of NTSR1 protein structure from homology modeling. (**A**) Ramachandran Plot of the modeling structure. The rationality of

stereochemistry parameters of NTSR1 modeling 3D structure is revealed by the number of residues in most favoured (red), additional allowed (yellow), generously allowed (light yellow) and disallowed regions (white). (**B**) Homology modeling validation using ERRAT program. The overall quality factor is evaluated by rationality of non-bonded interactions between different atom types. (**C**) Homology modeling validation using VERIFY3D program. The scores of residues are determined through the compatibility of an atomic model (3D) with its own amino acid sequence (1D). (**D**) Homology modeling validation using ProSA program. Z-score value is calculated through interaction energy between amino acid residues in the 3D structure. The dark dot in the red circle represents the number of residues and Z-score value of NTSR1 structure, while the region colored blue and light blue represents structures obtained from NMR and X-ray. Homology modeling is considered reasonable when Z-score value of the target protein are distributed within the graph range plotted by the Z-score values of these known proteins. (**E**) Homology modeling validation using ProQ program. LGscore>4 & MaxSub>0.8 is considered extremly good model, and LGscore>1.5 & MaxSub>0.1 is considered fairly good model.

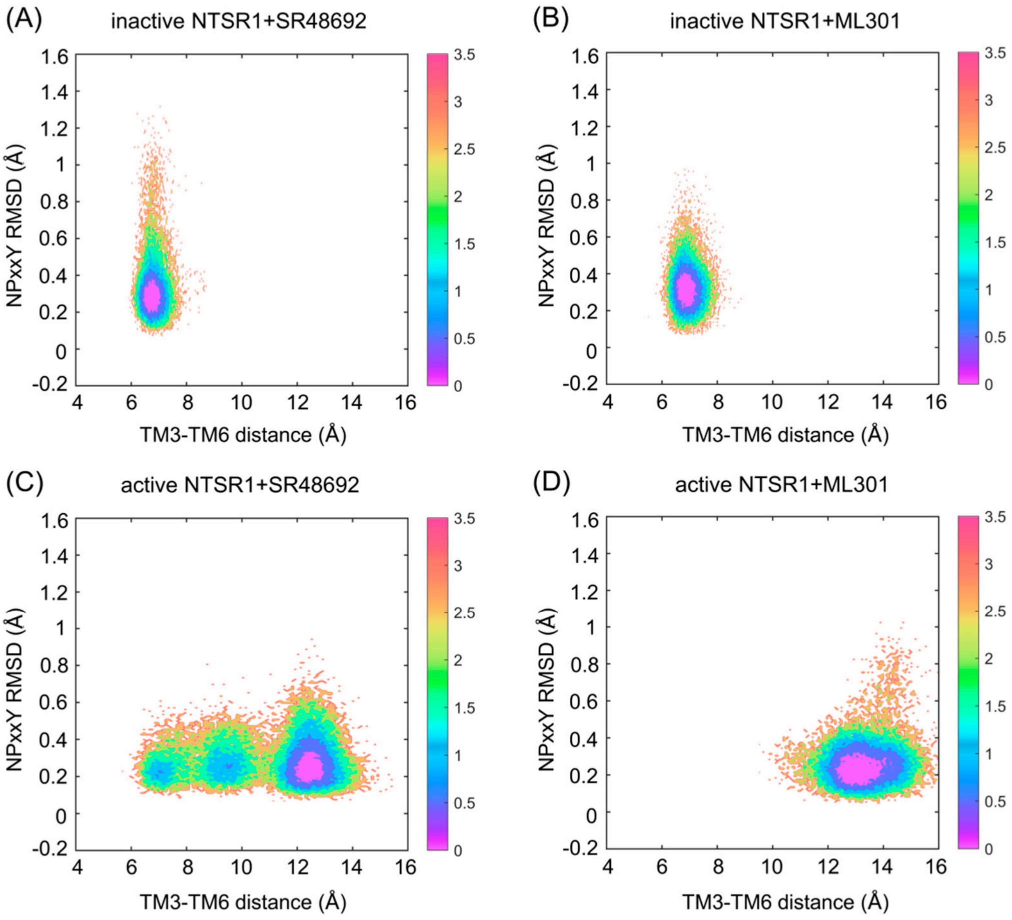

Figure S2. The analogical free energy landscape of the inactive NTSR1+SR48692 system (**A**), the inactive NTSR1+ML301 system (**B**), the active NTSR1+SR48692 system (**C**) and the active NTSR1+ML301 system (**D**) are shown by simulation trajectory projection. CV1: TM3-TM6 distance (evaluated by the distance between the center of mass of R166$^{3.50}$ in TM3 and V302$^{6.34}$ in TM6), CV2: NPxxY RMSD (evaluated by RMSD of non-symmetric side-chain atoms of residues N360$^{7.49}$ to Y364$^{7.53}$). Color scale on the right is evaluated through density.

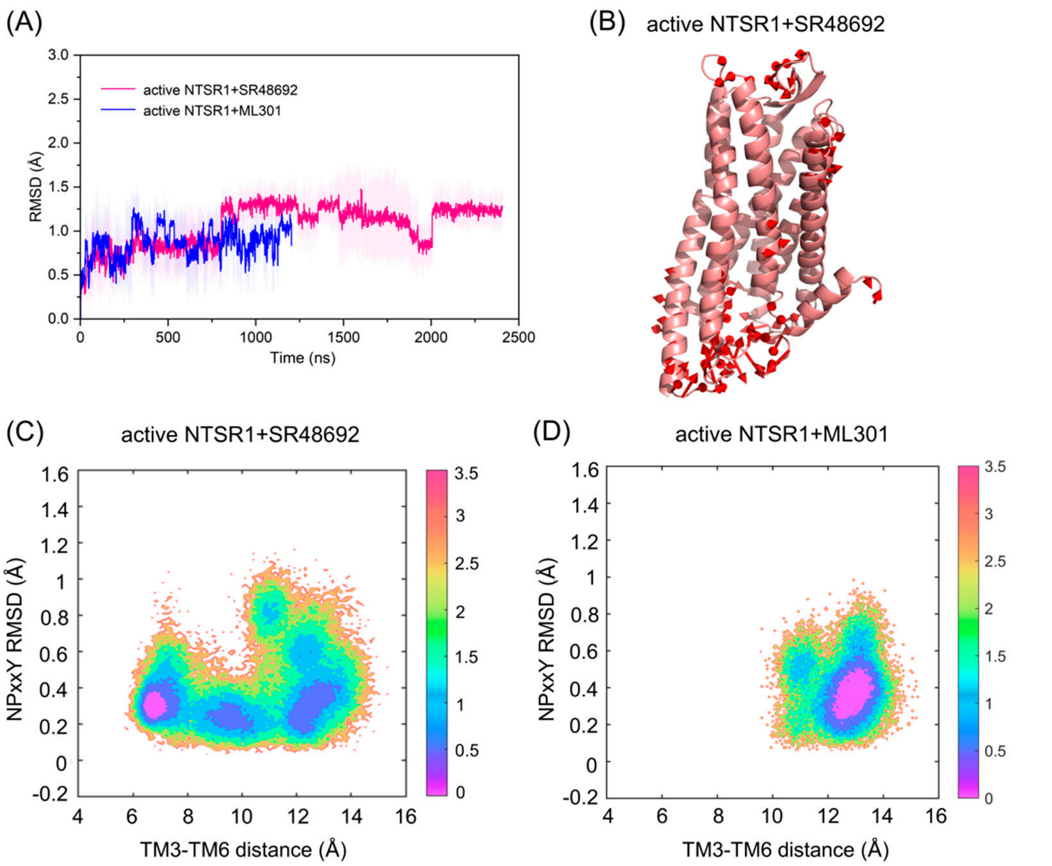

Figure S3. (**A**) The RMSD value of ligands in all simulations in the active NTSR1+SR48692 system (pink curve) and the active NTSR1+ML301 system (blue curve). (**B**) The principal pattern of motion of the active NTSR1+SR48692 system. The sizes and lengths of the red arrows are proportional to the amplitude of motions. The free energy landscapes of the active NTSR1+SR48692 system (**C**) and the active NTSR1+ML301 system (**D**) in cMD simulation. Collective variable 1 (CV1): TM3-TM6 distance (evaluated by the distance between the center of mass of R166$^{3.50}$ in TM3 and V302$^{6.34}$ in TM6), CV2: NPxxY RMSD (evaluated by RMSD of non-symmetric side-chain atoms of residues N360$^{7.49}$ to Y364$^{7.53}$). The unit of free energy values is kcal/mol. Color scale on the right is evaluated through free energy.

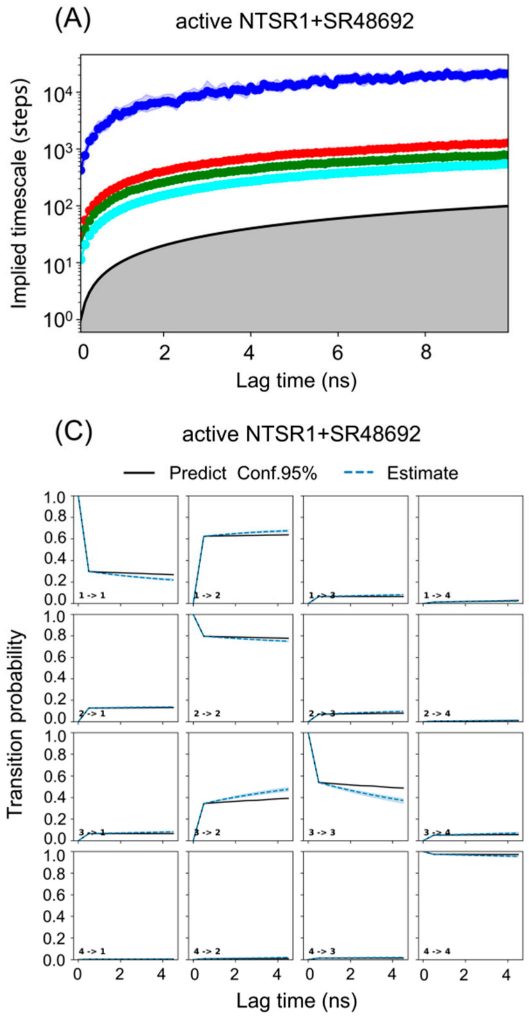

Figure S4. Implied timescale test for MSMs in the active NTSR1+SR48692 system (**A**) and the active NTSR1+ML301 system (**B**) at different lag times. Blue, red, green, and cyan lines show the timescale τ1, τ2, τ3 and τ4. Black line represents x=y in logarithmic coordinates, which separates the area where the dynamics of the processes is resolvable (white) from the non-resolvable area (grey). Chapman-Kolmogorov test of metastable states for the active NTSR1+SR48692 system (**C**) and the active NTSR1+ML301 system (**D**). The black solid estimate lines are the transition probability predicted by MSMs, while the blue dotted predict lines are practical transition probability observed in trajectories.

Table S1. Frequency of Y319$^{6.51}$-F353$^{7.42}$ and F353$^{7.42}$-R148$^{3.32}$ interaction in the representative trajectories in the active NTSR1+SR48692 system and the active NTSR1+ML301 system.

	Y319$^{6.51}$-F353$^{7.42}$ frequency	F353$^{7.42}$-R148$^{3.32}$ frequency
active NTSR1+SR48692 (active state)	62.4%	33%
active NTSR1+SR48692 (intermediate state)	75.3%	36%
active NTSR1+SR48692 (inactive state)	1.9%	17.9%
active NTSR1+ML301	48.3%	57.3%
